# Intravitreal Ranibizumab for Stage IV Proliferative Sickle Cell Retinopathy: A First Case Report

**DOI:** 10.1155/2014/682583

**Published:** 2014-11-23

**Authors:** Panagiotis G. Mitropoulos, Irini P. Chatziralli, Efstratios A. Parikakis, Vasileios G. Peponis, Georgios A. Amariotakis, Marilita M. Moschos

**Affiliations:** ^1^Ophthalmiatrion Athinon, 26 Eleftheriou Venizelou Street, 10672 Athens, Greece; ^2^Eye CU Center, 8 Naiadon Street, 11634 Athens, Greece; ^3^1st Department of Ophthalmology, University of Athens, G. Gennimatas Hospital, 154 Mesogeion Street, 11527 Athens, Greece

## Abstract

*Purpose*. To present the case of a 27-year-old male patient with stage IV proliferative sickle cell retinopathy, treated with one intravitreal injection of ranibizumab, showing regression of the neovascularization and no recurrence at the 9-month follow-up. *Methods*. A 27-year-old male patient presented with blurred vision and floaters in the right eye since three days. His best corrected visual acuity was 6/18. Ophthalmological examination and fluorescein angiography revealed proliferative sickle cell retinopathy stage IV with vitreous hemorrhage and sea fan neovascularization, as well as ischemic areas at the temporal periphery. *Results*. The patient was treated with one intravitreal injection of ranibizumab, presenting improvement in the visual acuity from 6/18 to 6/6, resolution of vitreous hemorrhage, and regression of the neovascularization. Additionally, he underwent scatter laser photocoagulation at the ischemic areas. At the 9-month follow-up there was no recurrence, while no adverse effects were noticed. *Conclusions*. Intravitreal ranibizumab may be a useful adjunct to laser photocoagulation in the management of proliferative sickle cell retinopathy and may permit some patients to avoid pars plana vitrectomy for vitreous hemorrhage.

## 1. Introduction

Sickle cell disease is the most common genetic disease in the world [1, 2]. Its pathophysiology is complex and not limited to abnormalities of the erythrocytes but can be related to metabolic pathways, including endothelial activation, inflammation, nitric oxide bioavailability, oxidative stress, and regulation of the adhesiveness of blood cells [3–5]. The sickle cell hemoglobinopathy with the greatest number of clinical implications is the SS type, while the SC type has rare systemic effects, although it is usually associated with the most severe and potentially blinding retinal manifestations [4–6]. Specifically, patients with sickle cell disease may present a great deal of retinal changes, such as hemorrhages, salmon patch, iridescent bodies, black sunburst, exudates, angioid streaks, and retinal vascular abnormalities, including tortuous vessels, microaneurysms, chorioretinal infarction, occlusion of the peripheral retinal vasculature, and consequent ischemia, leading to proliferative retinopathy, usually complicated with vitreous hemorrhages or retinal detachment [4, 5, 7].

In cases of proliferative retinopathy in sickle cell disease, vascular endothelial growth factor (VEGF) has been considered to play a key role in the formation of new blood vessels from the existing vasculature, as it appears to have a major role in ocular neovascularization in different capillary beds [8, 9]. Of note, VEGF has been found to be expressed and upregulated by ischemia and subsequent hypoxia in a great variety of in vitro and in vivo models [9]. Furthermore, it has been shown to increase vascular permeability, resulting in leakage of the dye on fluorescein angiography from the sea fan neovascularization [4, 10]. As a result, therapeutic strategies directed against VEGF are being investigated for many ocular diseases, giving a new perspective in the treatment armamentarium of ophthalmologists.

Interestingly enough, intravitreal ranibizumab (Lucentis; Novartis International AG, Basel, Switzerland) has been successfully used in the regression of retinal neovascularization due to proliferative diabetic retinopathy, ischemic retinal vein occlusion, retinopathy of prematurity, and Coats' disease or in cases of choroidal neovascularization due to age-related macular degeneration, myopia, and other neovascular eye diseases [11–17]. In light of the above, herein we presented the case of a man with stage IV proliferative sickle cell retinopathy, including sea fan neovascularization and vitreous hemorrhage, treated with one intravitreal injection of ranibizumab, showing regression of the neovascularization and no recurrence at the 9-month follow-up.

## 2. Case Description

A 27-year-old male patient presented with blurred vision and floaters in the right eye for three days with no improvement. His ophthalmological history was clear, while his medical history included sickle cell SC type hemoglobinopathy.

**Figure 1:**
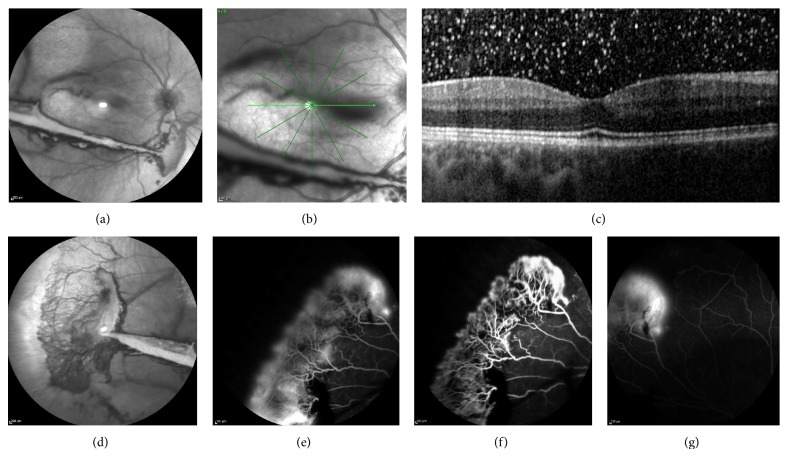
(a) Infrared fundus photo, showing vitreous hemorrhage in the right eye; (b, c) optical coherence tomography, showing no macular abnormalities; (d) infrared fundus photo, showing sea fan neovascularization at the temporal periphery; (e–g) fluorescein angiography, showing leakage from the neovascularization and ischemia in the periphery.

**Figure 2:**
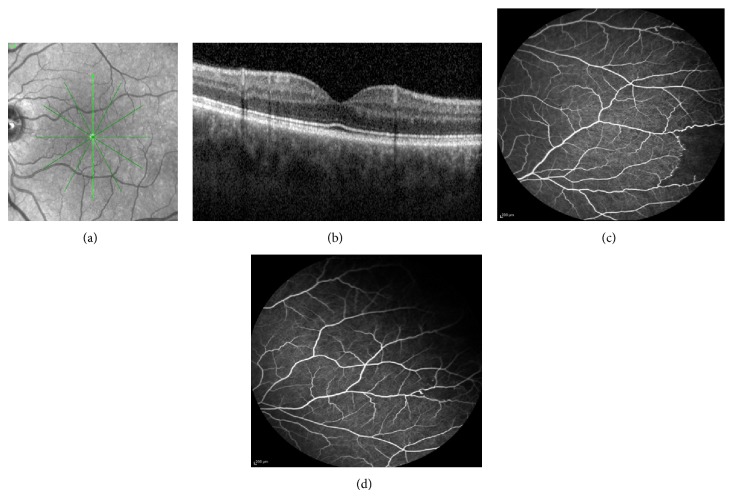
(a, b) Infrared fundus photo and optical coherence tomography in the left eye, totally normal; (c, d) fluorescein angiography, showing small ischemic areas at the temporal periphery of the left eye.

At presentation, his best corrected visual acuity (BCVA) was 6/18 in the right eye and 6/6 in the left eye, intraocular pressure was 13 mmHg in both eyes, and anterior segment slit-lamp examination was also unremarkable in both eyes. Dilated fundoscopy revealed vitreous hemorrhage in the right eye (stage IV proliferative sickle cell retinopathy) and normal left eye. The patient was counselled on the nature of the problem and referred to the medical retina department of our hospital. A fluorescein angiography was performed to evaluate the extent of retinopathy, confirming leakage from the sea fan neovascularization in the right eye, with ischemia in the periphery (Figure 1). In the left eye, only small ischemic areas in the temporal periphery were detected (Figure 2). Optical coherence tomography (OCT) was normal in both eyes (Figures 1 and 2).

Treatment alternatives were discussed with the patient and he was offered an off-label intravitreal ranibizumab injection in the right eye. Written informed consent was obtained from the patient for off-label use of ranibizumab, after explaining the potential complications of such a treatment. After placement of a sterile lid speculum and topical 5% iodine povidone, an intravitreal injection of 0.5 mg ranibizumab was administered in the right eye 4 mm posterior to the limbus using a 30-gauge needle.

**Figure 3:**
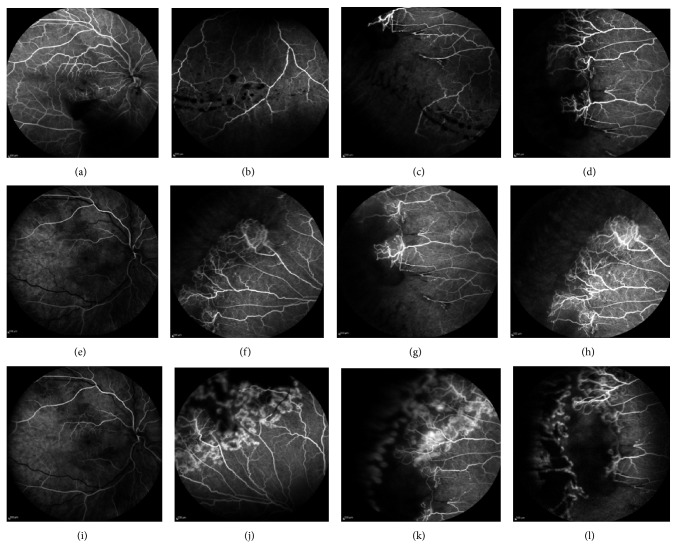
(a–d) Fluorescein angiography, showing improvement of vitreous hemorrhage and slight regression of neovascularization, but presence of ischemic areas, one week after intravitreal ranibizumab injection; (e–h) fluorescein angiography, showing total absorption of vitreous hemorrhage, regression of neovascularization and ischemia at the periphery one month after intravitreal ranibizumab injection; (i–l) fluorescein angiography, showing laser photocoagulation spots in the previous ischemic areas, no recurrence of vitreous hemorrhage, and regression of neovascularization compared to baseline.

**Figure 4:**
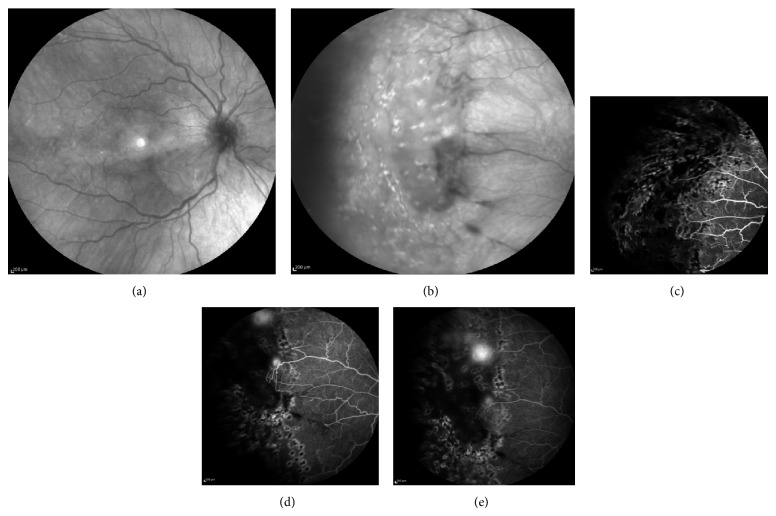
(a, b) Infrared fundus photo of the right eye, showing no vitreous hemorrhage and presence of fibrotic tissue at the site of previous neovascularization, as well as presence of laser photocoagulation spots in the periphery; (c–e) fluorescein angiography, showing coverage of previous ischemic areas with scatter laser photocoagulation and improvement of sea fan neovascularization, with only slight leakage hyperfluorescence in the photocoagulated area.

One week after injection, his BCVA was 6/9 in the right eye and fundus examination demonstrated improvement of vitreous hemorrhage, as well as regression of the sea fan neovascularization, confirmed by fluorescein angiography (Figure 3). One month after injection, the BCVA was 6/6 in both eyes, the vitreous hemorrhage was totally absorbed, and the retinal neovascularization further regressed (Figure 3). Scatter laser photocoagulation was also applied to the area of nonperfusion in the temporal periphery of the right eye. Three months later, there was no recurrence of the neovascularization, as it is depicted on fluorescein angiography (Figure 3) and the BCVA was 6/6, remaining stable at the 9-month follow-up (Figure 4), while no adverse events were observed.

## 3. Discussion

To our knowledge, this is the first case report of retinal neovascularization due to sickle cell disease, regressed by one intravitreal injection of ranibizumab. The treatment of sickle cell retinopathy remains controversial and depends on the stage of retinopathy. According to Goldberg et al., stage I consists of the irreversible vascular occlusion in the peripheral retina, leading to a remodeling of the vascular network, which forms new vascular arcades similar to normal ones. When arteriovenous communications in the boundary between the vascular and ischemic retina developed, the retinopathy is characterized as stage II. In these two stages, no treatment is needed, as it does not prevent the formation of sea fan neovascularization and it is thought that spontaneous resolution occurs in about 32% due to autoinfarction. Stage III is defined by the presence of neovascularization usually at the boundaries between the nonperfused and perfused areas of the retinal periphery, which have been referred as “sea fan” neovascularization, because of their resemblance to* Gorgonia flabellum*. Historically, sea fan neovascularizations have been treated in a variety of ways, such as diathermy, cryotherapy, and argon/xenon photocoagulation. The latter has been the most widely used treatment modality for proliferative sickle cell retinopathy stage III and nowadays consists mainly of sectorial scatter laser photocoagulation. Although sea fan neovascularizations are small and typically asymptomatic, they can rupture into the vitreous, resulting into vitreous hemorrhage (stage IV). Repeated vitreous hemorrhages produce cicatricial changes with the formation of glial bands into the vitreous, whose potential contraction may lead to retinal detachment (stage V). Surgical treatment with pars plana vitrectomy is needed for stage IV and V, although observation for about 6 months until vitreous hemorrhage is absorbed can be also advised [4, 5, 7, 18].

Nowadays, in the era of anti-VEGF agents, intravitreal injections of anti-VEGF could be a treatment alternative to proliferative sickle cell retinopathy. Siqueira et al. reported a case of a 36-year-old man with proliferative sickle cell retinopathy, including vitreous hemorrhage in the right eye, treated with an intravitreal injection of bevacizumab (1.5 mg/0.06 mL), as the patient had refused to undergo vitrectomy for the vitreous hemorrhage. His visual acuity presented improvement and there was regression of the neovascularization at the one-month follow-up, when photocoagulation of the ischemic areas was performed. The authors suggested that intravitreal injection of bevacizumab could be used as an adjunct to photocoagulation in the management of proliferative sickle cell disease and may preclude vitrectomy for some patients [19]. Accordingly, Shaikh described the case of a 32-year-old male patient with sickle cell disease, who presented with vitreous hemorrhage and was treated with intravitreal injection of 1.25 mg bevacizumab. His BCVA was improved from 20/40 to 20/20, the vitreous hemorrhage was resolved, and the neovascularization regressed, presenting no recurrence at the 6-month follow-up [20]. Contrary to Siqueira et al. and Shaikh, Babalola reported no resolution of vitreous hemorrhage and development of hyphema after intravitreal injection of bevacizumab for sickle cell retinopathy in a 25-year-old male patient, while in the other eye regression of stage III sea fan neovascularization was remarked [21]. Our case is the first one, using intravitreal ranibizumab for the treatment of stage IV sickle cell retinopathy, showing promising results. However, as the original incentive for neovascularization, which is capillary closure and peripheral retinal hypoxia, has probably not been redressed, laser therapy may still be indicated.

It is worthy to note that sickle cell retinopathy is typically asymptomatic until complications occur, such as vitreous hemorrhage and retinal detachment; therefore, meticulous periodic eye examination is needed. The role of fluorescein angiography has been established, revealing areas of leakage due to neovascularization, as well as ischemic regions. Spectral domain-OCT may also reveal atrophy of ganglion cells, of the inner nuclear layer and of Mueller cells due to chronic ischemia [4, 22]. In our patient, OCT was totally normal in both eyes.

In conclusion, intravitreal ranibizumab may be a useful adjunct to laser photocoagulation in the management of proliferative sickle cell retinopathy and may permit some patients to avoid pars plana vitrectomy for vitreous hemorrhage. Although our finding is promising, it requires additional investigation. Further studies should include a dose-response treatment to demonstrate the safety and efficacy of intravitreal ranibizumab for proliferative sickle cell retinopathy.
